# Micro-spatial distribution of malaria cases and control strategies at ward level in Gwanda district, Matabeleland South, Zimbabwe

**DOI:** 10.1186/s12936-017-2116-1

**Published:** 2017-11-21

**Authors:** Tawanda Manyangadze, Moses J. Chimbari, Margaret Macherera, Samson Mukaratirwa

**Affiliations:** 1 Department of Public Health Medicine, School of Nursing and Public Health, University of KwaZulu-Natal, Durban, South Africa; 2 Geography Department, Faculty of Science, Bindura University of Science Education, Bag 1020, Bindura, Zimbabwe; 3 Department of Environmental Science and Health, Faculty of Applied Sciences, National University of Science and Technology, Ascot, P O Box AC 939, Bulawayo, Zimbabwe; 4 School of Life Sciences, University of KwaZulu-Natal, Durban, South Africa

**Keywords:** Cluster detection, Malaria hotspots, Geographically weighted Poisson regression (GWPR) model, Malaria pre-elimination phase

## Abstract

**Background:**

Although there has been a decline in the number of malaria cases in Zimbabwe since 2010, the disease remains the biggest public health threat in the country. Gwanda district, located in Matabeleland South Province of Zimbabwe has progressed to the malaria pre-elimination phase. The aim of this study was to determine the spatial distribution of malaria incidence at ward level for improving the planning and implementation of malaria elimination in the district.

**Methods:**

The Poisson purely spatial model was used to detect malaria clusters and their properties, including relative risk and significance levels at ward level. The geographically weighted Poisson regression (GWPR) model was used to explore the potential role and significance of environmental variables [rainfall, minimum and maximum temperature, altitude, Enhanced Vegetation Index (EVI), Normalized Difference Vegetation Index (NDVI), Normalized Difference Water Index (NDWI), rural/urban] and malaria control strategies [indoor residual spraying (IRS) and long-lasting insecticide-treated nets (LLINs)] on the spatial patterns of malaria incidence at ward level.

**Results:**

Two significant clusters (p < 0.05) of malaria cases were identified: (1) ward 24 south of Gwanda district and (2) ward 9 in the urban municipality, with relative risks of 5.583 and 4.316, respectively. The semiparametric-GWPR model with both local and global variables had higher performance based on AICc (70.882) compared to global regression (74.390) and GWPR which assumed that all variables varied locally (73.364). The semiparametric-GWPR captured the spatially non-stationary relationship between malaria cases and minimum temperature, NDVI, NDWI, and altitude at the ward level. The influence of LLINs, IRS and rural or urban did not vary and remained in the model as global terms. NDWI (positive coefficients) and NDVI (range from negative to positive coefficients) showed significant association with malaria cases in some of the wards. The IRS had a protection effect on malaria incidence as expected.

**Conclusions:**

Malaria incidence is heterogeneous even in low-transmission zones including those in pre-elimination phase. The relationship between malaria cases and NDWI, NDVI, altitude, and minimum temperature may vary at local level. The results of this study can be used in planning and implementation of malaria control strategies at district and ward levels.

## Background

Malaria remains one of the biggest health problems within the tropical region despite the improvements in malaria control programmes at a global scale. There were approximately 212 million cases of infection and 429,000 malaria-related deaths in 2015; and more than 90% of these cases occurred in sub-Saharan Africa [1]. In Zimbabwe, malaria continues to be a major public health threat with an estimated over half of the population of 13.5 million at risk of contracting malaria [2]. However, by 2010, Zimbabwe had managed to reduce malaria incidence to 45 malaria cases per 1000 inhabitants per year thereby surpassing the Abuja 2010 set target of 68 cases per 1000 inhabitants [3]. Consistent with the national trend, Gwanda district located in the Matabeleland South Province in Zimbabwe has progressed to malaria pre-elimination phase. A malaria pre-elimination capacity assessment study conducted in Matabeleland South Province in 2011 reported malaria positivity rates of 8% and *Anopheles* larvae scoop of four for Gwanda district [4]. Malaria control in Gwanda district is mainly through indoor residual spraying (IRS), use of long-lasting insecticidal nets (LLINs) and larviciding [5].

Malaria transmission is heterogeneous at varying geographical scales even in the malaria pre-elimination zones [6]. Usually malaria endemicity levels, and especially in low incidence areas, malaria tends to cluster in ‘hotspots’ and ‘hot’ populations that become sources of continued infection [7]. Malaria hotspots have close spatial associations with vector-breeding habitats, and in certain ‘high-risk’ sub-sets of the population, having higher exposure to vector-breeding habitat: ‘hot-pops’ [8]. Active and timely identification of these hotspots and related factors is important for effective malaria control [7]. Malaria heterogeneity in time and space has also been attributed to risk factors, including altitude, climate, environmental parameters, and socio-economic factors [6, 7]. Rainfall, temperature and altitude are key factors in determining the habitat suitability of malaria vectors, including *Anopheles arabiensis* which is common in Zimbabwe, as well as determining malaria incidences [9–12]. In most tropical African countries, high habitat suitability of malaria vectors such as *An. arabiensis* mostly translates into increased malaria incidences [11]. Mabaso et al. [12] used a model to analyse the spatial and temporal role of climate in inter-annual variation of malaria incidence in Zimbabwe for the period 1988–1999 and their study demonstrated that mean values of temperature, rainfall and vapour pressure are strong predictors of malaria incidence. Gwitira et al. [13] also concluded that annual precipitation, precipitation of the wettest month, isothermality and temperature seasonality combined with altitude, are key predictors of *An. arabiensis* habitat suitability in Zimbabwe. In their study, the habitat suitability was significantly and positively correlated with recorded malaria incidences. Based on their observations they inferred that high malaria cases would be expected in areas of high vector habitat suitability. However, they also noted the need to consider malaria interventions in the study region in order to draw meaningful conclusions about the relationship between mosquito habitat suitability and malaria incidence.

Accurately assessing the local risk of transmission is fundamental for the development of malaria control programmes. Differences in malaria transmission exist, not just between different regions but also at local level [14–16]. As Gwanda district is moving towards malaria elimination phase a better understanding of the distribution of malaria cases/incidence at local scale is essential. In this regard, it is critical to understand the key factors for determining variability of malaria cases at micro-spatial scale for improving malaria control strategies in cases of relapses or outbreaks.

An exploratory study to investigate the relationship between the above-mentioned factors (rainfall, temperature, altitude, and other factors, including vegetation cover and wetness) at local scale is required. This paper reports on the spatial distribution of malaria incidence in 2015 based on health facility cases and related risk factors, in order to strengthen control measures in the pre-elimination phase of Gwanda district, Matabeleland South Province, Zimbabwe.

## Methods

### Study area

**Fig. 1:**
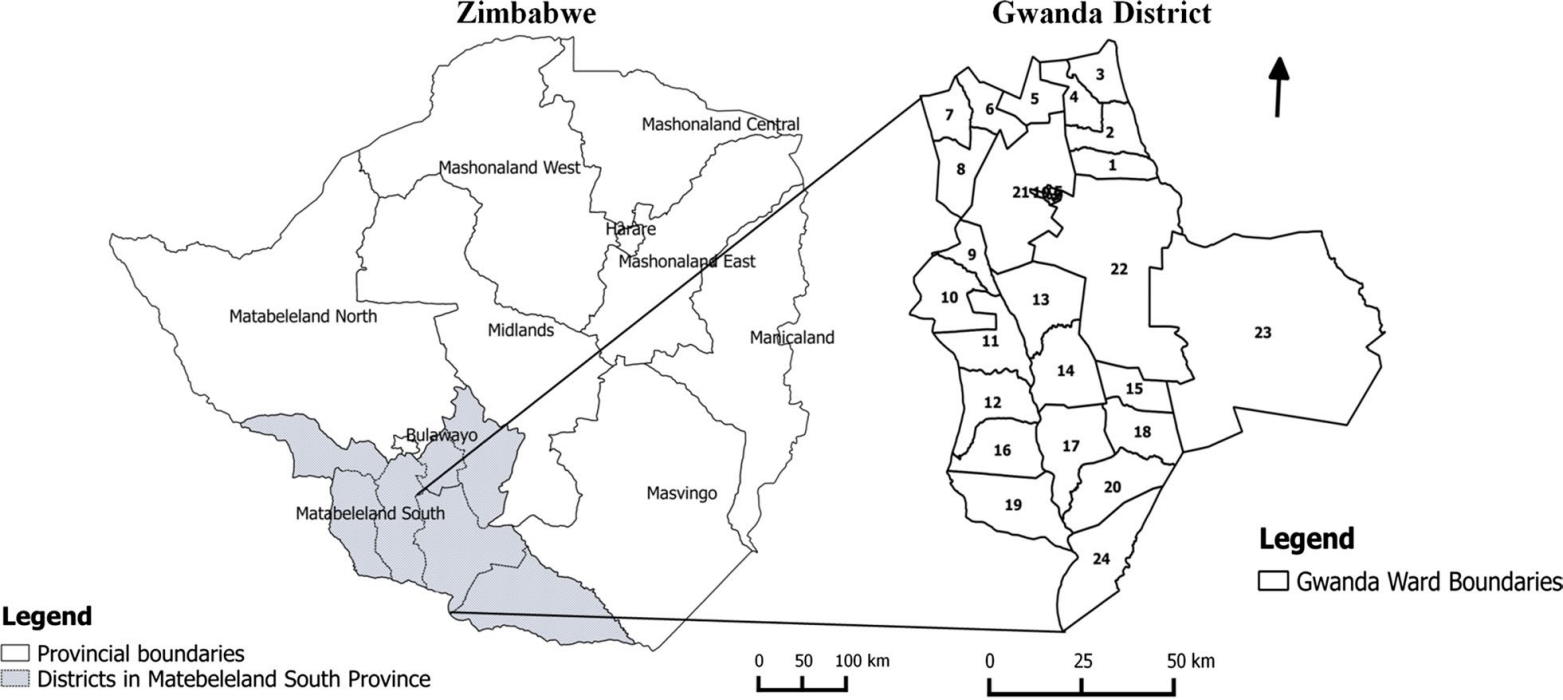
Gwanda district, Matabeleland South Province, Zimbabwe

Zimbabwe experiences seasonal and spatial variation in malaria transmission that is related to the country’s rainfall pattern [12]. The malaria peak transmission season in Zimbabwe is between February and April. This study was conducted in Gwanda district, Matabeleland South Province, Zimbabwe (Fig. 1). Gwanda district receives annual rainfall of lower than 700 mm, and that has necessitated the construction of dams and establishment of irrigation schemes to improve peoples’ livelihoods as many of them rely on subsistence agriculture. However, this has also resulted in increased exposure and vulnerability of people to malaria. Gwanda recorded the second highest incidence of malaria and mortality due to malaria in Matabeleland South Province in the period 2009–2013 [5].

Malaria control measures in Gwanda district include IRS; the use of LLINs and larviciding [5]. The planning of these control measures is done at district level and implemented at ward level. A ward is an administrative area under a district that consists of an average of 10 villages with each village comprised of an average of 100 households [5]. Gwanda has 24 and 10 rural and urban wards, respectively. Wards implement different malaria control programmes and also experience environmental conditions that could determine the spatial and distribution of malaria incidence/cases. The district implements IRS in some wards and the LLNs in other wards. This is a provincial decision where the areas with higher prevalence use IRS while those with lower prevalence use LLNs. The idea is to finally stop the use of IRS and focus on personal protection as the prevalence goes down. Given the variations across wards there is need to understand the transmission dynamics at ward level to improve the overall control programme to reach the elimination phase.

### Data collection

Mapping malaria in low-transmission settings is a challenge due to low incidence and malaria transmission tends to concentrate in hot spots and hot pops (populations that maintain malaria transmission) [17]. Data from health facilities (passive surveillance) provide a means of detecting hot spots of transmission [18, 19]. In this regard malaria cases recorded at health facilities in Gwanda district for 2015 were considered in the analysis in this study. Cases and their corresponding Global positioning systems (GPS) coordinates for the households were obtained from District Health Information System version 2 (DHIS2) through Ministry of Health and Child Care, Zimbabwe. These cases from health facilities had been parasitologically confirmed through rapid diagnostic tests (RDTs) or microscopy, and were complete both spatially and temporally. The DHIS2 system was implemented in mid-2014, hence 2015 had a complete dataset by the time of data collection (August 2016). The estimated total population per ward was used to calculate the incidence per ward [20].

The environmental/ecological/climatic/topographic factors considered include rainfall, temperature, wetness, vegetation cover. Table 1 shows data on these variables indicating the sources. These variables were accessed and processed (including the calculation of averages per ward) through the IRI data library portal [21]. The seasonal variation and within ward heterogeneity were not considered in this study.

**Table 1:** Source of environmental data used at ward level for 2015 in Gwanda district, Matabeleland South Province, Zimbabwe

Variables	Minimum	Mean	Maximum	Data source	Resolution	Reference
Rainfall	16.990	21.731	24.090	UCBS^c^	5 km	ftp://ftp.chg.ucsb.edu/pub/org/chg/products/CHIRPS-2.0/
T_max_	37.374	39.490	43.029	USGS^d^	1 km	http://modis.gsfc.nasa.gov/data/dataprod/mod11.php
T_min_	13.979	15.958	17.990	USGS^d^	1 km	http://modis.gsfc.nasa.gov/data/dataprod/mod11.php
Altitude	653.24	918.465	1071.89	NASA^e^	1 km	http://www2.jpl.nasa.gov/srtm
NDVI^a^	0.301	0.366	0.442	USGS^d^	250 m	http://modis.gsfc.nasa.gov/data/dataprod/mod13.php
EVI^b^	0.172	0.209	0.243	USGS^d^	250 m	http://modis.gsfc.nasa.gov/data/dataprod/mod13.php
NDWI	− 0.040	0.024	0.133	USGS^d^	250 m	Calculated based on MODIS reflectance

*T*_*max*_ maximum temperature, *T*_*min*_ minimum temperature

^a^Normalised Difference Vegetation Index

^b^Enhanced Vegetation Index

^c^University of California Santa Barbara

^d^United States geological survey

^e^National Aeronautics and Space Administration

Malaria control measures considered in this study include the coverage of LLINs and IRS. The data on malaria control measures per ward in Gwanda district and the extent of coverage were obtained from Ministry of Health and Child Care, Zimbabwe. The data were presented as percentage coverage or percentage population protected (%). It was also considered whether the ward was rural or urban.

### Spatial analysis

The Poisson purely spatial model [22] was used to determine the malaria hot spots/clusters and the geographically weighted Poisson regression (GWPR) model [23] to examine the potential role of environmental variables and malaria control strategies on the spatial patterns of malaria cases recorded at health facilities and aggregated to a ward level. The GPS coordinates from DHIS2 were plotted and overlaid on the Gwanda ward boundaries to obtain the number of cases per ward. Malaria incidence was calculated as number of cases per ward divided by the total population per ward multiplied by 1000. The Poisson purely spatial model in SatScan spatial scan statistic [22] was used to detect the malaria hot spots or clusters. In this model, the numbers of malaria cases follow a heterogeneous Poisson process [23, 24]. The spatial scan statistic is a local cluster test [24] which has been widely used in spatial-epidemiological studies to detect local clusters with statistically significant elevated risk of infectious diseases [24–27]. The input data for this model included the number of malaria cases per ward, the approximate total population per ward, and the centroid coordinates for each ward. Considering that Gwanda is in the pre-elimination phase characterized by low number of cases, the maximum population at risk was set not to exceed 2% of the total population. This improves the precision in detecting local clusters since the default value of 50% of the population at risk is more likely to produce clusters of no practical use [25, 28].

The GWPR model [23] in GWR4.09 package (GWPR development team) was used to explore the local variation of malaria cases in relation to the environmental factors and coverage of control measures at local scale (ward level). Exploratory disease mapping and local cluster tests have been used for identifying areas with statistically significant high risks—hot spots or clusters. GWPR has proved to be very effective for measuring the spatially varying association between possible factors and disease risk. This allows the development of disease control measures targeting specific population groups that are most at risk in specific locations across the landscape [25, 29–31].

In this study it was assumed that the numbers of malaria cases in each ward follow independent Poisson distributions. Hence, *Y*_*i*_ denote the number of malaria cases observed in ward *i* in Gwanda district, *i* = 1,…, 34. GWPR allows the examination of the spatially varying coefficients over space and the semiparametric GWPR uses the local and global terms. The GWPR and semiparametric GWPR models are defined in Eqs. 1 and 2, respectively:

1 $$ y_{i} \sim Poisson\left( {N_{i} \exp \left( {\mathop \sum \limits_{k} \beta_{k} (u_{i} ,v_{i} )x_{i,k} } \right)} \right) $$

2 $$ y_{i} \sim Poisson\left( {N_{i} \exp \left( {\mathop \sum \limits_{k} \beta_{k} (u_{i} ,v_{i} )x_{i,k} +  \mathop \sum \limits_{l} \gamma_{l} z_{l,i}  } \right)} \right) $$

where *y*_*i*_ *, x*_*i,k*_, and *N*_*i*_ denote, respectively, dependent variable (the total number of malaria cases), *k*th independent variable including the constant term and the offset variable [population size at risk (ward population) in the ward *i*]. (*u*_*i*_, *v*_*i*_) is the geographic coordinate of the centroid of the *i*th ward (the location of *i*). The coefficients *β*_*k*_ (*u*_*i*_, *v*_*i*_) are assumed to be smoothly varying conditional on their location. *z*_*l*,*i*_ is the *l*th independent variable with a fixed coefficient *γ*_*l*_.

Three models were computed: (1) a global model that assumes that the process accounting for the disease is spatially constant throughout the study area; (2) a local model that assumed all environmental factors and control measures vary locally; and, (3) a model including local and global variables. The adaptive bi-square kernel for geographically weighting was used in this study. This is suitable for clarifying local extends for model fitting and keeping constant the number of areas to be included in the kernel [32]. The adaptive bi-square kernel is also more suitable for when one seeks a definitive local extent for model fitting [33]. The golden search was used as a bandwidth selection method. Considering that the sample size was small, the minimum and maximum bandwidth ranges were defined as 30 and 34 respectively in GWR4.09. The golden search helps to automatically reach or obtain the optimum bandwidth size based on the Akaike information criterion (AICc) [34].

The local model computed a spatial weights matrix based on the assumption of spatial autocorrelation. This means that the individual observation was influenced by the surrounding observations and the extent of this influence was inversely related to distance [35]. The local model provided locally varying parameter estimates, standard errors, as well as the respective pseudo t values as described by Nakaya et al. [23]. A pseudo t value less than − 1.96 or greater than + 1.96 indicates a p value  <  0.05. The weighting function, the bandwidth size of the matrix and the best model regarding different independent variable sub-sets were based on the small sample size bias corrected AICc comparison as suggested by Alves et al. [33]. The smaller AICc indicates a better model performance. If the difference between AICc is larger than 2, the model with lower AICc is selected [36]. The coefficients and the percent deviance explained (measure of local goodness-of-fit and is a type of pseudo-R^2^ [37]) were only showed for the best performing model based on the AICc. These coefficients were mapped using QGIS 2.2.0. The wards with significant coefficients were also shown on each local coefficient map.

### Statistical analysis

Before performing the GWPR model, correlation analyses was conducted in Excel 2007 to ensure that the variables (Table 2) were not highly correlated. Strong multicollinearity can impair the model and produce artificial and erroneous effects [38]. The variance inflation factor (VIF) was used as an indicator of multicollinearity. Generally, VIF value of greater than 4–10 is regarded as severe multicollinearity [39–41]. Hence, the lower limit (> 4) was used as a cut-off in this study as also used by Ehlkes et al. [35].

**Table 2:** Characteristics of malaria clusters for year 2015 in Gwanda District, Matabeleland South Province, Zimbabwe

	Cluster 1	Cluster 2
Wards included	Ward 24 (rural—South)	Ward 9 (urban—North)
Population	2699	2539
Number of cases	7	6
Expected cases	1.48	1.39
Annual cases/1000	2.59	2.36
Observed/expected	4.74	4.32
Relative risk	5.13	4.61
Log likelihood ratio	5.583	4.316
p value	0.0024	0.012

## Results

### Distribution of malaria in 2015

**Fig. 2:**
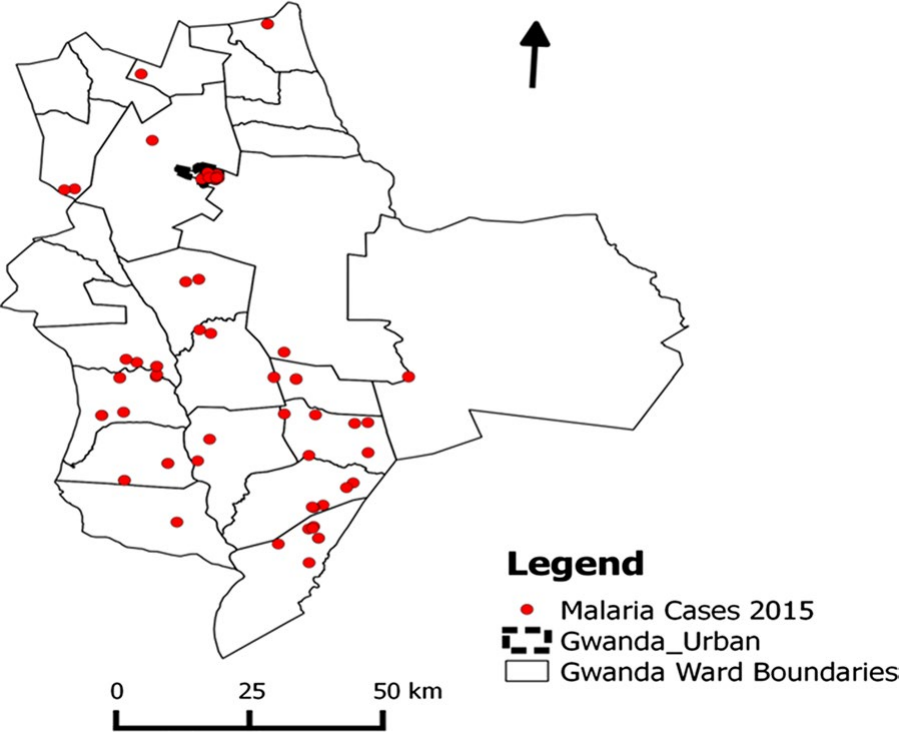
Malaria cases per ward in Gwanda district, Matabeleland South Province, Zimbabwe (2015)

**Fig. 3:**
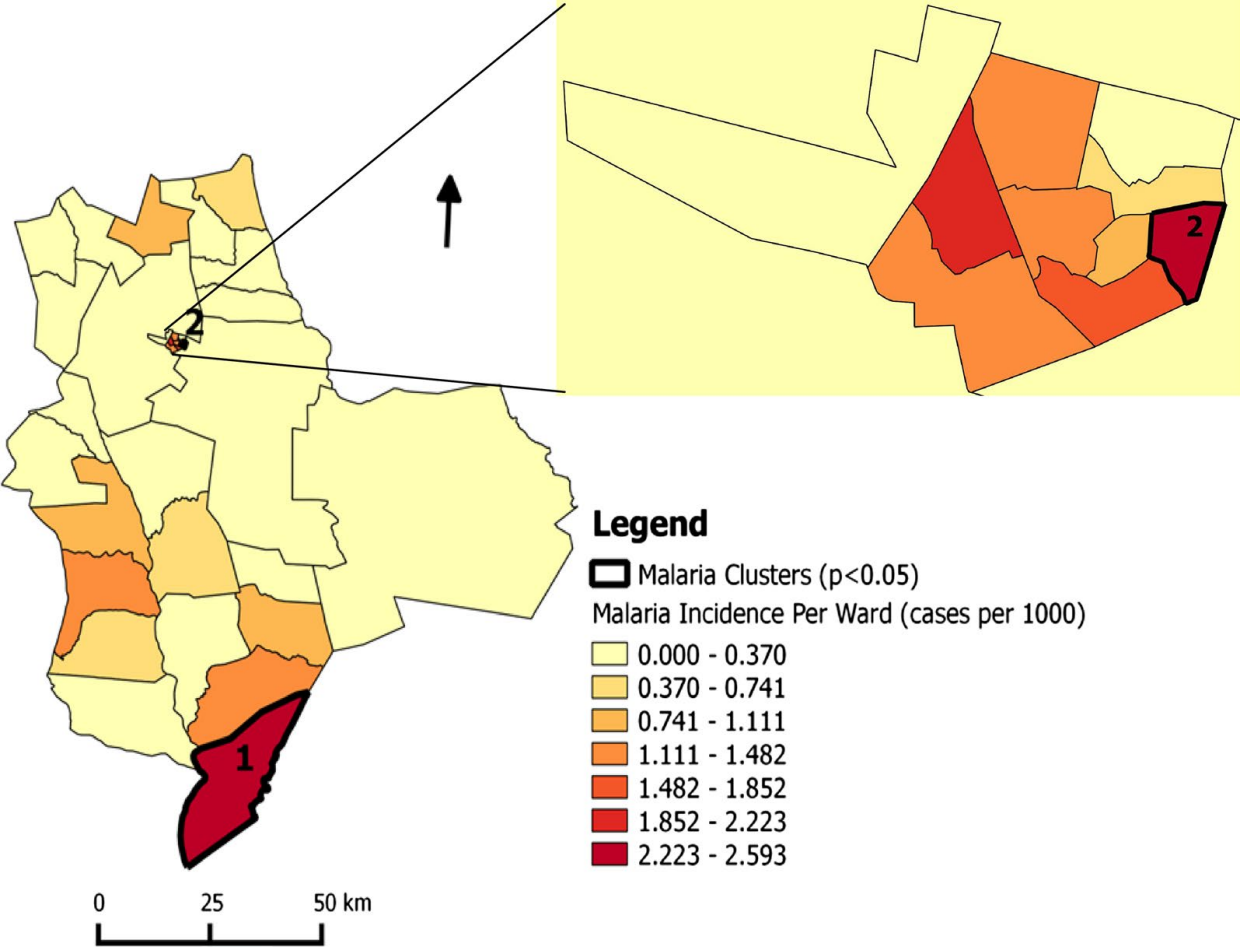
Malaria incidence and clusters per ward in Gwanda district, Matabeleland South Province in Zimbabwe (2015)

A total of 75 cases in a total population of approximately 137,105 in Gwanda was recorded in 2015. This translates to overall annual malaria incidence of 0.547 per 1000 inhabitants. The cases were distributed in the wards as shown in Fig. 2. The incidence per ward ranged from 0 to 2.59 per 1000 inhabitants (Fig. 3). Two significant malaria clusters for year 2015 were detected in Gwanda district. The properties of these clusters (Fig. 3) are shown in Table 2.

**Fig. 4:**
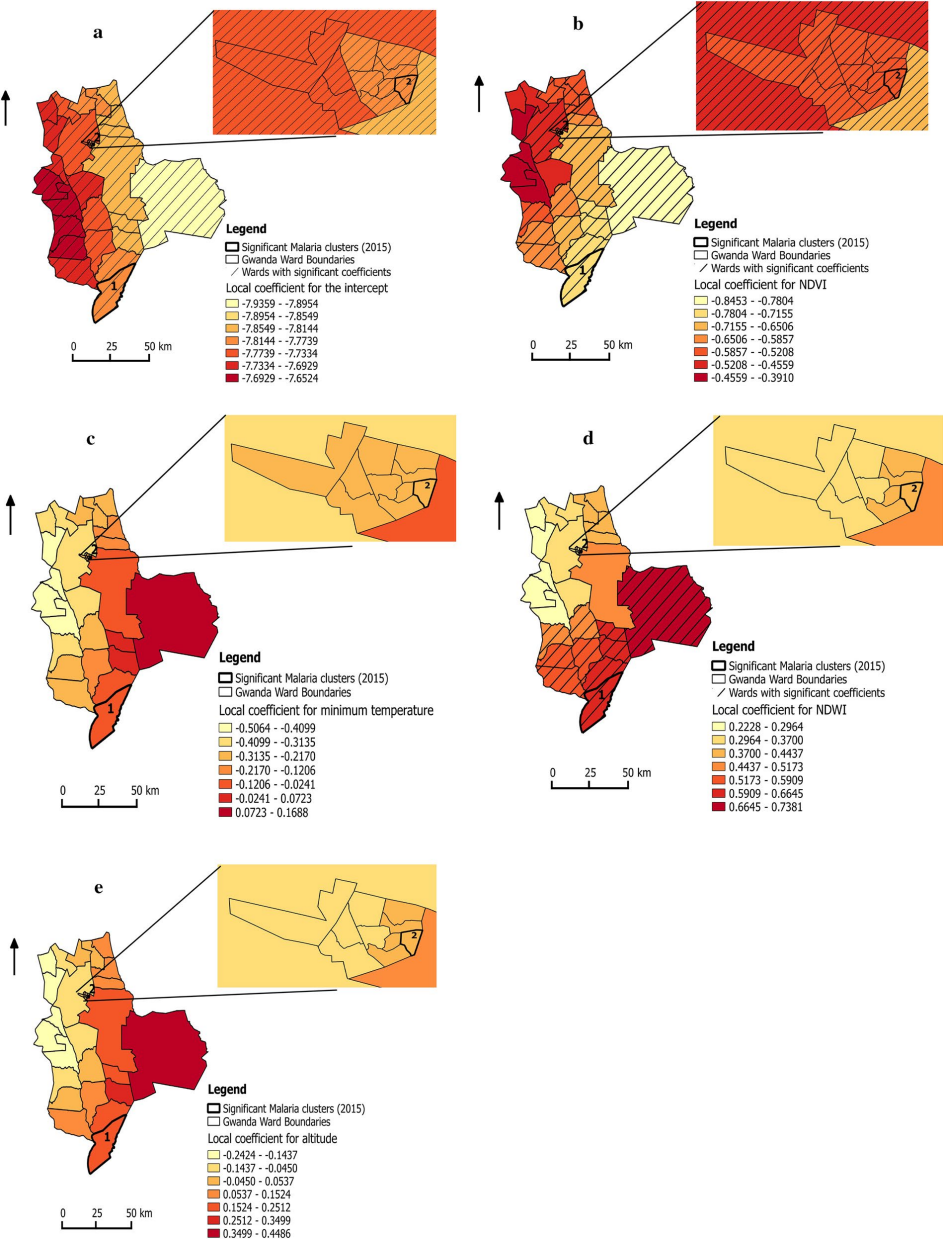
Variation of local coefficient estimates from GWPR **a** intercept, **b** Normalized Difference Vegetation Index (NDVI), **c** minimum temperature, **d** Normalized Difference Water Index (NDWI), **e** altitude in Gwanda district, Matabeleland South Province, Zimbabwe (2015)

The clusters were detected in both rural and urban wards; cluster 1 (ward 24) and cluster 2 (ward 9), respectively. Rural cluster had higher relative risk compared to the urban cluster (Table 2). The coefficients of the variables from the s-GWPR model in rural and urban areas including the clusters are shown in Fig. 4.

### Local variation of exploratory variables in determining the heterogeneity of malaria incidence

The semiparametric-GWPR model with mixed variables (after local to global) had an AICc of 70.882 and a percent deviance explained of 0.529. This model used 34 (all samples) as the optimum bandwidth based on the adaptive kernel and golden search for bandwidth selection. This model performed better than other models (Table 3) and was further used to explore the local variation of the exploratory variables in relation to malaria cases.

**Table 3:** Comparison of model performances based on AICc

Model	AICc
Global regression	74.390
GWPR (before local to global)	73.364
Semiparametric-GWPR (after local to global)	70.882

After local to global variable selection, only three variables were moved to global. These variables include LLINs, IRS and rural or urban with the following estimated coefficients: 0.844, − 0.171, and − 0.873, respectively. The influence of these variables did not vary across the study area and remained in the models as global terms. The other exploratory variables were varying at ward level (Fig. 4); the summary of their coefficients is shown in Table 4.

**Table 4:** Summary of the coefficients of the locally varying variables based on the best s-GWPR model

Coefficients	Minimum	Lower quartile	Median	Upper quartile	Maximum
Intercept	− 7.936	− 7.795	− 7.776	− 7.729	− 7.652
Minimum temperature	− 0.506	− 0.346	− 0.278	− 0.191	0.169
NDVI^a^	− 0.845	− 0.622	− 0.541	− 0.516	− 0.391
NDWI^b^	0.223	0.347	0.374	0.509	0.738
Altitude	− 0.242	− 0.088	− 0.011	0.099	0.449

^a^Normalised Difference Vegetation Index

^b^Enhanced Vegetation Index

The semiparametric-GWPR captured the spatially non-stationary relationships between malaria cases and some of the exploratory variables (minimum temperature, Normalized Difference Vegetation Index (NDVI), Normalized Difference Water Index (NDWI), and altitude) at the ward level. The coefficients of these variables and the intercept in the GWPR varied spatially, suggesting the effects of the exploratory variables were different between wards. Gwanda district is not entirely homogeneous as it was shown by wide ranges of estimated coefficients for NDWI, NDVI, minimum temperature and altitude. NDWI (proxy for surface water or wetness) estimated coefficients showed positive association with malaria cases per ward and was significant in the rural wards located to the southern and southeastern part of the study area. Altitude and minimum temperature estimated coefficients ranged from negative to positive but not significant. The estimated NDVI coefficients were negative over the whole study area and significant in all urban wards and most of the rural wards. The intercept coefficients were negative and significant across the whole study area.

### Multicollinearity

The VIF for all the variables was ranging from 0.822 to 19.378. Three variables showed high correlation with other variables. These include rainfall, maximum temperature and EVI with VIF of 10.509, 19.378 and 4.929, respectively; hence these variables were not considered in the model.

## Discussion

Results of this study have shown that the distribution of annual cases or incidence of malaria is heterogeneous even in the malaria pre-elimination zones, as observed in other studies [6–8]. Differences exist not just between different regions but also at local level [14–16]. Identifying the malaria clusters (areas with elevated number of cases) is critical in developing or improving malaria control strategies at local scale. In this study, clusters were detected in rural and urban areas. This indicates that malaria incidence could be high or above average in both rural and urban settings in Gwanda district. However the cluster had a higher relative risk compared to the urban cluster. These clusters may point to the areas that need immediate attention in terms of preparation and implementation of the disease control strategies.

Heterogeneity of malaria cases is driven by a variety of ecological, biological and sociological factors [6]. As noted by Ehlkes et al. [35] most studies assume homogeneous influence of exploratory variables [42–45] but this may not always be most appropriate [23]. In this study, the analysis showed that assuming that other variables vary at local level substantially improves the GWPR model performance. Allowing spatial heterogeneity within the regression model allows clearer interpretation regarding the true nature of potential associations [35]. That could be because the LLINs are mostly distributed to the wards that are known to have higher incidences in Gwanda district and there was limited data regarding the actual use of these nets. When assessing the associations between environmental variables and malaria cases one must consider the pathways in which these variables under study lie [35]. For example, the environmental variables: minimum temperature, NDVI, altitude, NDWI which influence the malaria cases were considered in this study as they determine the abundance of mosquito or their breeding habitats. Malaria control strategies (IRS and LLINS coverage) per ward were also considered in this study. These factors tend to reduce the cases of malaria. The interaction between these factors and malaria cases may bring out unexpected results, defying the norms regarding the relationship between environmental factors and malaria. It has been established that transmission potential decreases as the altitude increases [2, 46, 47]. This was also noted in this study as altitude showed its expected negative relationship with malaria cases in some of the wards but also showed positive relationship in the wards to the southern part of Gwanda district. However, it was not significant in any of the wards. NDWI coefficients showed the expected positive association with malaria cases and were significant in the rural wards located in south and southeastern part of Gwanda district. Altitude and minimum temperature estimated coefficients ranged from negative to positive over Gwanda district. This indicates that GWPR successfully captured the spatially non-stationary of these factors and how the global model can be misleading since averaging these local effects reveals a single impact assumed to hold across all regions [33]. The estimated NDVI coefficients were negative over the study area, which was against the expectation.

The weak positive and strong negative correlation coefficients between environmental factors and malaria incidences in some of the wards could be due to the protection effect of malaria control factors, such as vector control methods including LLINs and IRS. These malaria interventions contribute significantly to the decline in malaria cases particularly in areas progressing towards malaria elimination [47]. Gwitira et al. [13] also noted that in cases where there is effective malaria control, there will be weak correlations between habitat suitability and malaria cases. This was observed in this study based on the proxies for land cover (NDVI), wetness (NDWI). However, there is need to consider mapping the land use and land cover types and relate to malaria cases at micro-scale. Changes in land use and land cover have also been found to be critical in determining the survival of *Anopheles* malaria vectors [35, 48]. Significant land cover and land use changes may lead to increase in the abundance of malaria vectors and consequently increased malaria transmission.

Geographically weighted regression (GWR) has shown its ability to handle the socio-economic variables in relation to disease transmission. For example, the semiparametric-GWR (s-GWR) managed to detect schistosomiasis hot spots based on socio-economic and environmental factors at household level in Ndumo area, uMkhanyakude in South Africa [35]. Active surveys are required to capture the data on socio-economic factors, including housing structure and entomological data. Previous studies showed that malaria transmission tends to be higher in houses built with mud and thatch than those with asbestos or iron sheets and are built using cement [9]. All these factors influence the spatial and temporal distribution of malaria incidence. The assessment of the effect of malaria control measures may need to be done in spatio-temporal modelling. The temporal aspect would be able to show the decrease or increase in malaria cases in relation to control measures and environmental factors over time. There was also no information regarding larviciding for 2015 and 2016. In the pre-elimination and elimination phases, interventions have to be targeted to entire villages or towns with higher malaria incidence until only individual episodes of malaria remain and become the centre of attention [49].

This study has shown the feasibility of using passive surveillance data from health facilities to map malaria cases and detect clusters. The passively identified health facility cases reflected malaria transmission levels in places where malaria cases tend to cluster at ward level as also noted by Rulisa et al. [7]. These cases were parasitologically confirmed using RDTs or microscopy and were complete both spatially and temporally. The clinical malaria cases were previously used in trend analysis of malaria transmission in relation to climatic and environmental factors in Tubu village, Botswana [50]. However, these data are usually incomplete [51], and future studies may consider approaches that adjust for health facility utilization and under-reporting [52, 53]. Sturrock et al. [17] noted that mapping malaria in low transmission settings is a challenge, given that as incidence drops, transmission concentrates in hot spots and hot pops. It is challenging to identify hot pops operationally because only a sub-set of febrile individuals may seek treatment at formal health facilities [53]. In low malaria transmission settings, the treatment-seeking patterns may be determined by individual immune status [54]. For example, the onset of fever in low-immunity populations may lead to presentation at peripheral health centres, while populations highly exposed (hot pops) are less likely to seek treatment [55]. These malaria hot spots may serve to perpetuate residual malaria transmission in low-transmission seasons and hinder efforts to eliminate malaria [56]. Active and timely identification of these hot spots and associated risk factors through active surveys is essential for targeting interventions to optimize malaria control [49].

This study presents an analysis of a single year of cross-sectional data that was temporally aggregated, hence the temporal dimension was not considered. Using only 1 year of data may only reflect where cases occurred during that single year, but not temporally stable areas of high malaria risk. This ignores and masks any seasonal pattern, which is highly important in areas approaching elimination. However the results of this study will inform further exploratory studies in pre-elimination zones also considering the amount of data which will be collected through DHIS2. The analysis in this study was also restricted by the limited number of wards in Gwanda district which impacted on sample size. Ward is the smallest political administrative unit with reliable data on population size. This might have impacted on the analysis as Paez et al. [57] advised that GWR may not be used in cases with sample size less than 160 as the small sample size issue could result in inaccurate estimates in statistical modelling [57–60]. However, Li et al. [61], noted that after considering the spatial heterogeneity in the county-level data, with sample size less than 160, the GWPR outperformed the traditional generalised linear models (GLM) in predicting fatal crashes in individual counties [61]. Therefore this study contributes to other GWR studies on disease and risk factors, most of which show that global statistical models may produce misleading results [62]. GWPR models are capable of capturing the spatial heterogeneity of phenomenon compared to global estimates/models [63]. The local coefficient maps in this study also show the magnitude, significance and direction of the relationships between malaria cases and exploratory variables. For local planning, such as district or ward (as in this case), the local GWR models seem to be more appropriate, since global models may not capture local changes or variation [63].

## Conclusions

This study has confirmed that malaria incidence is heterogeneous in low-transmission zones including those in pre-elimination phase. It explored local variations in the relationship between malaria cases and environmental and malaria control factors at ward level in Gwanda district, Matabeleland South Province, Zimbabwe. In 2015, Gwanda district had two significant clusters: ward 24 (rural) and 9 (urban). The NDVI and NDWI showed significant association with malaria cases in some of the wards in Gwanda district. Despite the tendency to underestimate malaria burden, routine data from health facilities are helpful in reflecting the spatial distribution of malaria, especially in low malaria incidence settings. The results of this study can be used in planning and implementation of malaria control strategies at both district and ward levels.

## Abbreviations

IRS: indoor residual spraying
GWPR: geographically weighted Poisson regression
NDVI: Normalized Difference Vegetation Index
NDWI: Normalized Difference Water Index
EVI: Enhanced Vegetation Index
GPS: Global positioning system
LLIN: long-lasting insecticide-treated net
DHIS2: District Health Information System version 2
RDT: rapid diagnostic test
VIF: variance inflation factor
AICc: Akaike information criterion
UCSB: University of California Santa Barbara
USGS: US geological survey
NASA: National Aeronautics and Space Administration

## References

[1] WHO (2016). World malaria report 2015.

[2] Chikodzi D (2013). Spatial modelling of malaria risk zones using environmental, anthropogenic variables and geographical information systems techniques. J Geosci Geomat.

[3] WHO (2011). An information bulletin of the WCO Zimbabwe 2010.

[4] District Health Information (2016). Malaria reoprt 2016.

[5] Macherera M, Chimbari MJ, Mukaratirwa S (2017). Indigenous environmental indicators for malaria: a district study in Zimbabwe. Acta Trop.

[6] Pinchoff J, Henostroza G, Carter BS, Roberts ST, Hatwiinda S, Hamainza Bu (2015). Spatial patterns of incident malaria cases and their household contacts in a single clinic catchment area of Chongwe District, Zambia. Malar J.

[7] Rulisa S, Kateera F, Bizimana JP, Agaba S, Dukuzumuremyi J, Baas L (2013). Malaria prevalence, spatial clustering and risk factors in a low endemic area of Eastern Rwanda: a cross sectional study. PLoS ONE.

[8] Parker DM, Matthews SA, Yan G, Zhou G, Lee MC, Sirichaisinthop J (2015). Microgeography and molecular epidemiology of malaria at the Thailand–Myanmar border in the malaria pre-elimination phase. Malar J.

[9] Nkuo-Akenji T, Ntonifor NN, Ndukum MB, Kimbi HK, Abongwa EL, Nkwescheu A (2008). Environmental factors affecting malaria parasite prevalence in rural Bolifamba, South–West Cameroon. Afr J Health Sci.

[10] Adeola AM, Botai OJ, Olwoch JM, Rautenbach CJW, Adisa OM, Taiwo OJ (2016). Environmental factors and population at risk of malaria in Nkomazi municipality, South Africa. Trop Med Int Health.

[11] Lindsay SW, Parson L, Thomas CJ (1998). Mapping the ranges and relative abundance of the two principal African malaria vectors, *Anopheles gambiae* sensu stricto and *An. arabiensis*, using climate data. Proc R Soc London B.

[12] Mabaso MLH, Vounatsou P, Midzi S, Da Silva J, Smith T (2006). Spatio-temporal analysis of the role of climate in inter-annual variation of malaria incidence in Zimbabwe. Int J Health Geogr.

[13] Gwitira I, Murwira A, Zengeya FM, Masocha M, Mutambu S (2015). Modelled habitat suitability of a malaria causing vector (*Anopheles arabiensis*) relates well with human malaria incidences in Zimbabwe. Appl Geogr.

[14] Guthmann JP, Palacios A, Hall AJ (2002). Environmental factors as determinants of malaria risk. A descriptive study on the northern coast of Peru. Trop Med Int Health.

[15] Ageep TB, Cox J, Hassan MM, Knols BGJ, Benedict MQ, Malcolm CA (2009). Spatial and temporal distribution of the malaria mosquito *Anopheles arabiensis* in northern Sudan: influence of environmental factors and implications for vector control. Malar J.

[16] Djènontin A, Bio-Bangana S, Moiroux N, Henry MC, Bousari O, Chabi J (2010). Culicidae diversity, malaria transmission and insecticide resistance alleles in malaria vectors in Ouidah-Kpomasse-Tori district from Benin (West Africa): a pre-intervention study. Parasites Vectors.

[17] Sturrock HJW, Hsiang MS, Cohen JM, Smith DL, Greenhouse B, Bousema T (2013). Targeting asymptomatic malaria infections: active surveillance in control and limination. PLoS Med.

[18] Oduro AR, Bojang KA, Conway DJ, Corrah T, Greenwood BM, Schellenberg D (2011). Health centre surveys as a potential tool for monitoring malaria epidemiology by area and over time. PLoS ONE.

[19] Boulos MNK, Resch B, Crowley DN, Breslin JG, Sohn G, Burtner R (2011). Crowdsourcing, citizen sensing and sensor web technologies for public and environmental health surveillance and crisis management: trends, OGC standards and application examples. Int J Health Geogr.

[20] Zimbabwe National Statistics Authority (2013). Census 2012, provincial report. Matabeleland South.

[21] IRI data library. http://iridl.ldeo.columbia.edu/SOURCES/.

[22] Kulldorff M (1997). A spatial scan statistic. Commun Stat Methods.

[23] Nakaya T, Fotheringham AS, Brunsdon C, Charlton M (2005). Geographically weighted Poisson regression for disease association mapping. Stat Med.

[24] Tanser MF, Bärnighausen T, Cooke GS, Newell ML, Bärnighausen T (2009). Localized spatial clustering of HIV infections in a widely disseminated rural South African epidemic. Int J Epidemiol.

[25] Kauhl B, Heil J, Hoebe CJPA, Schweikart J, Krafft T, Dukers-Muijrers NHTM (2015). The spatial distribution of hepatitis C virus infections and associated determinants—an application of a geographically weighted poisson regression for evidence-based screening interventions in hotspots. PLoS ONE.

[26] Jennings JM, Curriero FC, Celentano D, Ellen JM (2005). Geographic identification of high gonorrhea transmission areas in Baltimore, Maryland. Am J Epidemiol.

[27] Alencar CH, Alberto NR, Barbosa JC, Kerr LRFS, Oliveira MLWDE, Heukelbach J (2012). Persisting leprosy transmission despite increased control measures in an endemic cluster in Brazil: the unfinished agenda. Lepr Rev.

[28] Chen J, Roth RE, Naito AT, Lengerich EJ, Maceachren AM (2008). Geovisual analytics to enhance spatial scan statistic interpretation: an analysis of US cervical cancer mortality. Int J Health Geogr.

[29] Lin CH, Wen TH (2011). Using geographically weighted regression (GWR) to explore spatial varying relationships of immature mosquitoes and human densities with the incidence of dengue. Int J Environ Res Public Health.

[30] Hu M, Li Z, Wang J, Jia L, Liao Y, Lai S (2012). Determinants of the incidence of hand, foot and mouth disease in China using geographically weighted regression models. PLoS ONE.

[31] Haque U, Scott LM, Hashizume M, Fisher E, Rashidul H, Yamamoto T (2012). Modelling malaria treatment practices in Bangladesh using spatial statistics. Malar J.

[32] Nakaya T. Geographically weighted regression (GWR) software. GWR 4.0. ASU GeoDa Center; 2014.

[33] Alves ATJ, Nobre FF, Waller LA (2016). Exploring spatial patterns in the associations between local AIDS incidence and socioeconomic and demographic variables in the state of Rio de Janeiro, Brazil. Spat Spatiotemporal Epidemiol.

[34] Manyangadze T, Chimbari MJ, Gebreslasie M, Mukaratirwa S (2016). Risk factors and micro-geographical heterogeneity of *Schistosoma haematobium* in Ndumo area, uMkhanyakude district, KwaZulu-Natal, South Africa. Acta Trop.

[35] Ehlkes L, Krefis AC, Kreuels B, Krumkamp R, Adjei O, Ayim-Akonor M (2014). Geographically weighted regression of land cover determinants of *Plasmodium falciparum* transmission in the Ashanti Region of Ghana. Int J Health Geogr.

[36] Ribeiro MC, Sousa AJ, Pereira MJ (2015). A coregionalization model to assist the selection process of local and global variables in semi-parametric geographically weighted poisson regression. Procedia Environ Sci.

[37] Nakaya T, Charlton M, Lewis P, Brunsdon C, Yao J, Fotheringham S. GWR4 user manual; 2014. http://geoinformatics.wp.st-andrews.ac.uk/download/software/GWR4manual.pdf. Accessed 20 Feb 2017.

[38] Wheeler D, Tiefelsdorf M (2005). Multicollinearity and correlation among local regression coefficients in geographically weighted regression. J Geogr Syst.

[39] Kennedy P (1998). A guide to econometrics.

[40] O’Brien RM (2007). A caution regarding rules of thumb for variance inflation factors. Qual Quant.

[41] Barbieri AF, Sawyer DO, Soares-filho BS (2005). Population and land use effects on malaria prevalence in the Southern Brazilian Amazon. Hum Ecol.

[42] Mushinzimana E, Munga S, Minakawa N, Li L, Feng C, Bian L (2006). Landscape determinants and remote sensing of anopheline mosquito larval habitats in the western Kenya highlands. Malar J.

[43] Dambach P, Machault V, Lacaux J, Vignolles C, Sié A, Sauerborn R (2012). Utilization of combined remote sensing techniques to detect environmental variables influencing malaria vector densities in rural West Africa. Int J Health Geogr.

[44] Stefani A, Roux E, Fotsing J, Carme B (2011). Studying relationships between environment and malaria incidence in Camopi (French Guiana) through the objective selection of buffer-based landscape characterisations. Int J Epidemiol.

[45] Taylor P, Mutambu SL (1986). A review of the malaria situation in Zimbabwe with special reference to the period 1972–1981. Trans R Soc Trop Med Hyg.

[46] Ebi KL, Chan N, Hartman J, Mcconnell J, Schlesinger M, Weyant J (2015). Climate suitability for stable malaria transmission in Zimbabwe under different climate change scenarios. Clim Chang.

[47] Meyrowitsch DW, Pedersen EM, Alifrangis M, Scheike TH, Malecela MN, Magesa SM (2011). Is the current decline in malaria burden in sub-Saharan Africa due to a decrease in vector population. Malar J.

[48] Stresman GH, Kamanga A, Moono P, Hamapumbu H, Mharakurwa S, Kobayashi T (2010). A method of active case detection to target reservoirs of asymptomatic malaria and gametocyte carriers in a rural area in Southern Province, Zambia. Malar J.

[49] WHO (2007). Malaria elimination: a field manual for low and moderate endemic countries.

[50] Chirebvu E, Chimbari MJ, Ngwenya BN, Sartorius B (2016). Clinical malaria transmission trends and its association with climatic variables in Tubu Village, Botswana: a retrospective analysis. PLoS ONE.

[51] Gething PW, Noor AM, Gikandi PW, Ogara EAA, Hay SI, Nixon MS (2006). Improving imperfect data from health management information systems in Africa using space-time geostatistics. PLoS Med.

[52] Alegana VA, Atkinson PM, Lourenço C, Ruktanonchai NW, Bosco C, Erbach-Schoenberg E (2016). Advances in mapping malaria for elimination: fine resolution modelling of *Plasmodium falciparum* incidence. Sci Rep.

[53] Littrell M, Gatakaa H, Evance I, Poyer S, Njogu J, Solomon T (2011). Monitoring fever treatment behaviour and equitable access to effective medicines in the context of initiatives to improve ACT access: baseline results and implications for programming in six African countries. Malar J.

[54] Snow RW, Amratia P, Mundia CW, Alegana VA, Kirui VC, Kabaria CW, et al. Assembling a geo-coded repository of malaria infection prevalence survey data in Africa 1900–2014. Working paper in support of the Information or Malaria (INFORM) Project funded by the Department for International Development and The Wellcome Trust, UK. 2015.

[55] Snow RW, Marsh K (2002). The consequences of reducing transmission of *Plasmodium falciparum* in Africa. Adv Parasitol.

[56] Moonen B, Cohen JM, Tatem AJ, Cohen J, Hay SI, Sabot O (2010). A framework for assessing the feasibility of malaria elimination. Malar J.

[57] Páez A, Farber S, Wheeler D (2011). A simulation-based study of geographically weighted regression as a method for investigating spatially varying relationships. Environ Plan A.

[58] Wood GR (2002). Generalised linear accident models and goodness of fit testing. Accid Anal Prev.

[59] Lord D (2006). Modeling motor vehicle crashes using Poisson-gamma models: examining the effects of low sample mean values and small sample size on the estimation of the fixed dispersion parameter. Accid Anal Prev.

[60] Lord D, Mannering F (2010). The statistical analysis of crash-frequency data: a review and assessment of methodological alternatives. Transp Res Part A Policy Pract.

[61] Li Z, Wang W, Liu P, Bigham JM, Ragland DR (2013). Using geographically weighted Poisson regression for county-level crash modeling in California. Saf Sci.

[62] Leyk S, Norlund PU, Nuckols JR (2012). Robust assessment of spatial non-stationarity in model associations related to pediatric mortality due to diarrheal disease in Brazil. Spat Spatiotemporal Epidemiol.

[63] Pirdavani A, Brijs T, Bellemans T, Wets G. Spatial analysis of fatal and injury crashes in Flanders, Belgium: application of geographically weighted regression technique. In: The 92th annual meeting of transportation research board, Washington, DC. 2013.

